# Laparoscopic abdominal perineal rectal resection for rectal cancer with a horseshoe kidney using preoperative 3D-CT angiography: a case report

**DOI:** 10.1186/s12893-020-01032-y

**Published:** 2021-01-06

**Authors:** Jun-ichi Yoshizawa, Kuniyuki Gomi, Arano Makino, Ryo Hisamune, Sinsuke Sugenoya, Kou Shimada, Kiyotomi Maruyama, Motohiro Mihara, Shoji Kajikawa

**Affiliations:** 1 Department of Surgery, Suwa Red Cross Hospital, 5-11-11-50, Kogan-dori, Suwa, Nagano 392-8510 Japan; 2 Present Address: Department of Surgery, Ina Central Hospital, 1313-4, Koshirokubo, Ina, Nagano 396-8555 Japan

**Keywords:** Horseshoe kidney, 3D-CT, Rectal cancer

## Abstract

**Background:**

A horseshoe kidney is a congenital malformation involving the fusion of the bilateral kidneys and is often accompanied by anomalies of the ureteropelvic and vascular systems. When performing resection of colorectal cancer in a patient with horseshoe kidney, damage to the ureter or excessive renal arteries should be avoided. To achieve this purpose, comprehensive preoperative anatomical assessments and surgical planning are important. Here, we report a case of a laparoscopic abdominal perineal rectal resection for lower rectal cancer with a horseshoe kidney.

**Case presentation:**

A 79-year-old woman presented with bloody stool and was diagnosed with advanced lower rectal cancer, immediately above the rectal dentate line, without metastasis. A preoperative computed tomography (CT) scan revealed a horseshoe kidney, while a three-dimensional CT (3D-CT) angiography revealed aberrant excess renal artery from the aorta to the renal isthmus. The left ureter ran in front of the isthmus of the horseshoe kidney and presented calculus formation. Laparoscopic abdominal perineal rectal resection was performed with D3 lymph node dissection. During the operation, we mobilized the sigmoid colon mesentery via a medial approach and preserved the left ureter, the left gonadal vessels, and the hypogastric nerve plexus in the retroperitoneum in front of the horseshoe kidney.

**Conclusions:**

We report a rare case of rectal cancer surgery in a patient with a horseshoe kidney. We discuss the anatomical peculiarities of a horseshoe kidney, such as excess renal arteries, inferior vena cava, ureter, gonadal vessels, and nerves, that should be preserved according to the literature. We suggest that preoperative 3D-CT angiography is both useful for revealing the relationship between the vascular system and a horseshoe kidney and helpful when performing laparoscopic surgery for a left-sided colon and rectal cancer to avoid intraoperative injury.

## Background

A horseshoe kidney is a congenital malformation in which the bilateral kidneys are fused. It is likely the most common of all renal fusion anomalies, occurring in 0.25% of the population [1]. Patients with a horseshoe kidney often have abnormalities in the ureteral running and the vascular system, and it is necessary to pay attention to secondary damage during colorectal cancer surgery. Preoperative three-dimensional-computed tomography (3D-CT) angiography is effective for visualizing the relationship between the vascular structure and the horseshoe kidney without any secondary damage. We successfully performed laparoscopic abdominal perineal rectal resection with D3 lymph node dissection around the root of the inferior mesenteric artery (IMA) for lower rectal cancer with a horseshoe kidney.

## Case presentation

**Fig. 1:**
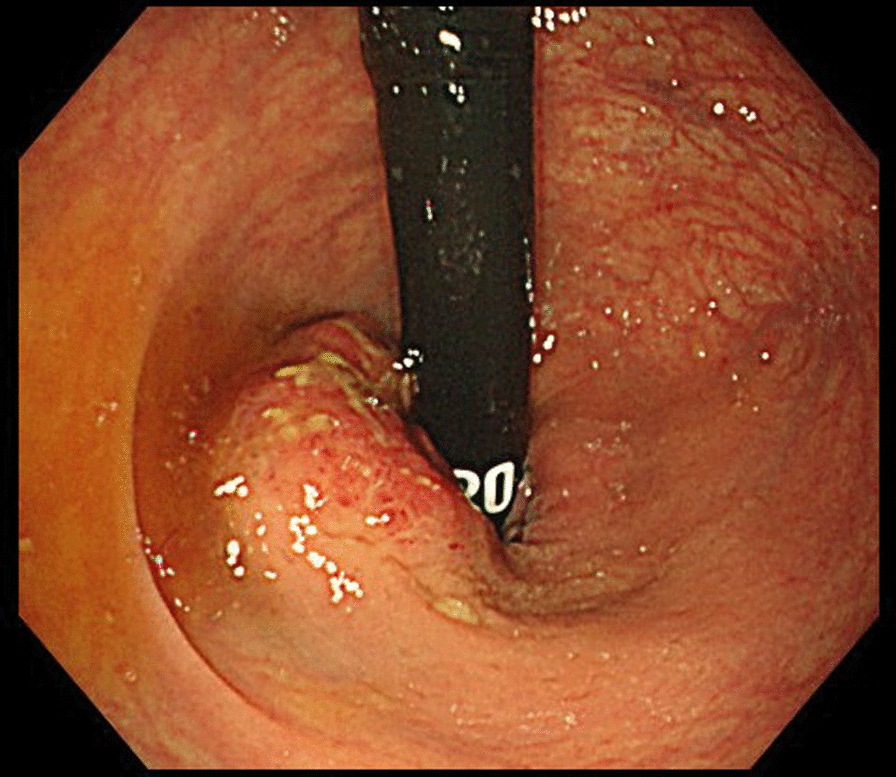
Lower endoscopic findings. Lower endoscopy revealed a semicircular type 3 lesion in the rectum just above the rectal dentition showing muscular infiltration

**Fig. 2:**
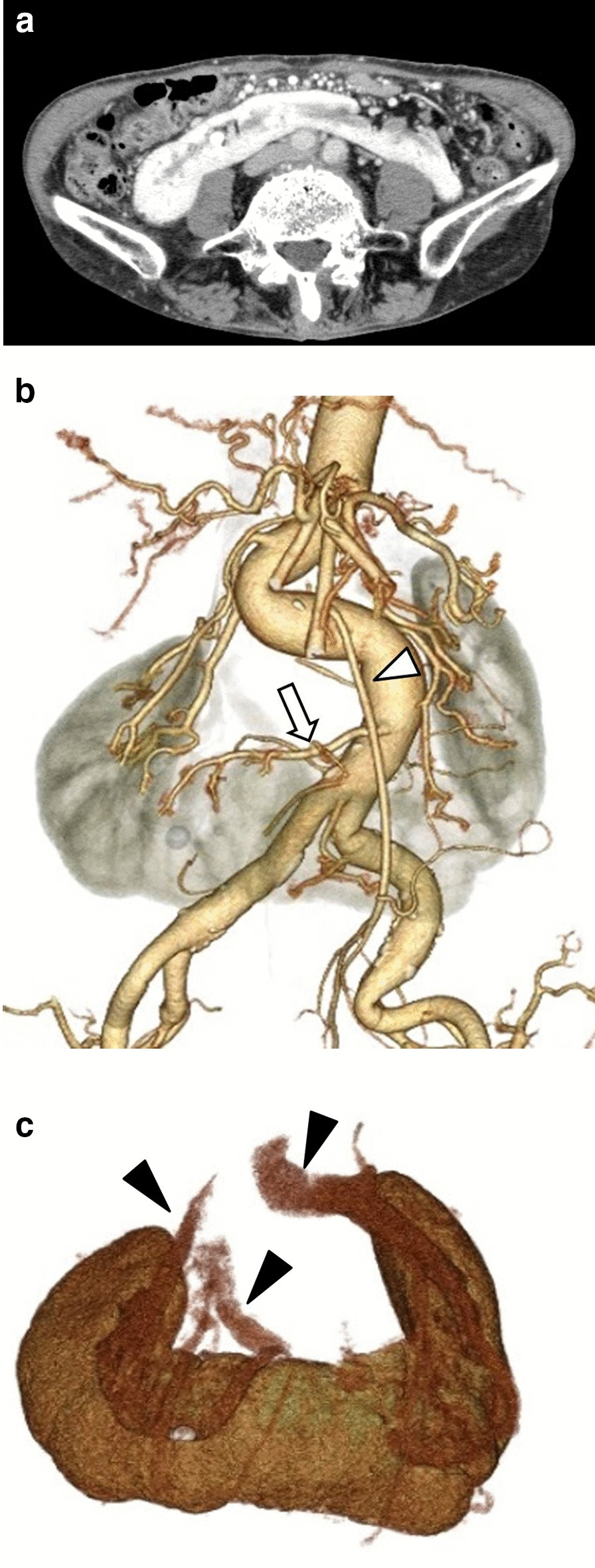
Computed tomography (CT) findings. **a** Abdominal enhanced CT showing the horseshoe kidney. **b**, **c** Three-dimensional CT (3D-CT) angiography showing an aberrant renal artery (arrow) from the aorta below the inferior mesenteric artery (white arrowhead) to the isthmus of the horseshoe kidney and a renal vein from (black arrowhead) the horseshoe kidney

A 79-year-old woman, presenting with bloody stool, underwent lower endoscopy, revealing a type 3 lesion with a semicircular cancer spread in the rectum, immediately above the rectal dentate line, showing muscular infiltration (T2) (Fig. 1). She was diagnosed with advanced rectal adenocarcinoma without lymph node (N0) or distant metastasis (M0). A preoperative CT scan incidentally revealed a horseshoe kidney (Fig. 2a), while a 3D-CT angiography revealed a meandered descending aorta, two renal arteries supplying each side of the kidney from the aorta, and an aberrant excess renal artery (Fig. 2b). The excess renal artery was supplying the surrounding of the isthmus and the left side of the lower horseshoe kidney branching from the aorta originating above the level of the isthmus. Additionally, 3D-CT angiography showed that the renal veins from the horseshoe kidney merged into the left and right renal veins, which merged into the inferior vena cava (IVC) (Fig. 2c). The left ureter ran in front of the isthmus of the horseshoe kidney with calculus formation in the ureter.

**Fig. 3:**
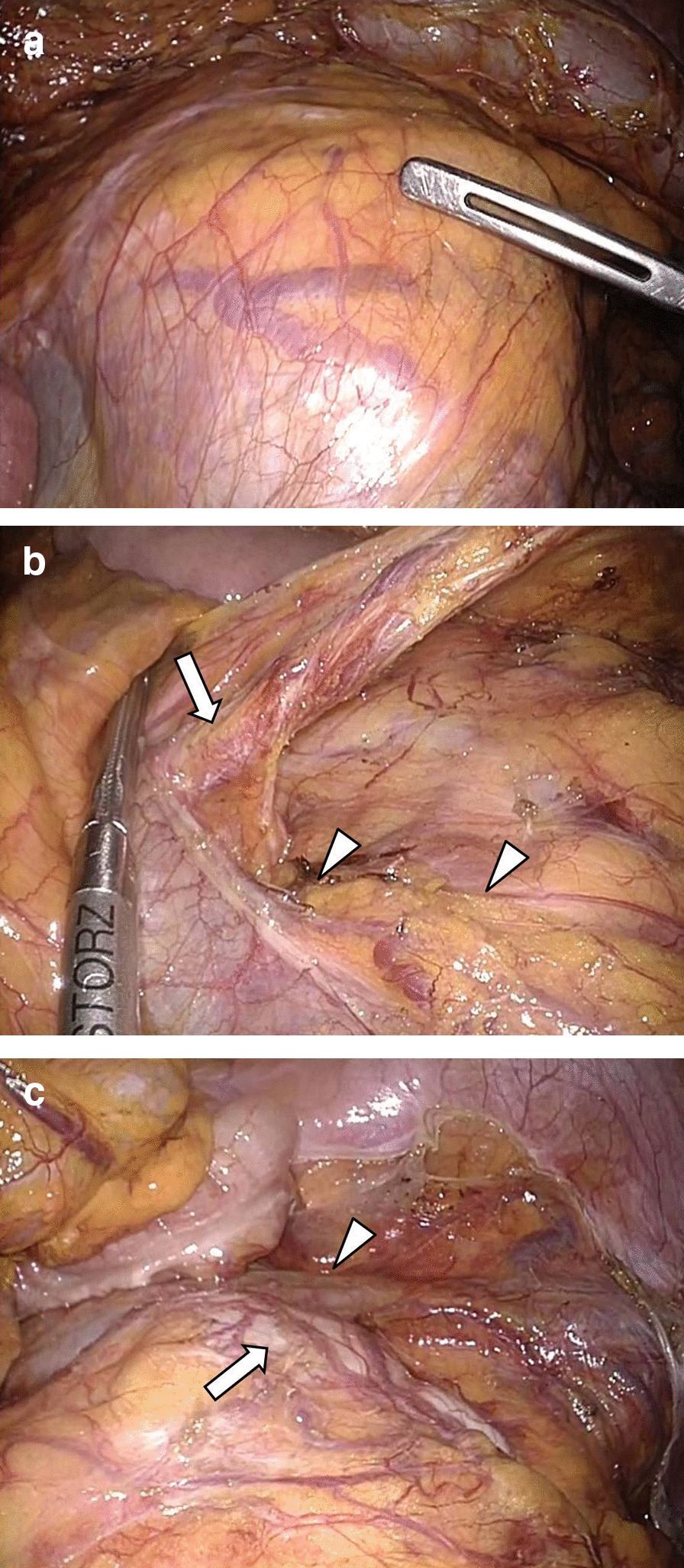
Surgical findings. **a** Retroperitoneal prominence due to the horseshoe kidney. **b** The inferior mesenteric artery root is on the temporal side of the renal isthmus (arrow), and the hypogastric nerve plexus is running craniocaudally on the front of the horseshoe kidney (arrowhead). **c** After dissecting the sigmoid mesocolon from the retroperitoneum, including the isthmus of the horseshoe kidney, the left ureter (arrow) and left gonadal vessels (arrowhead) were identified on the surface of the isthmus and preserved

**Fig. 4:**
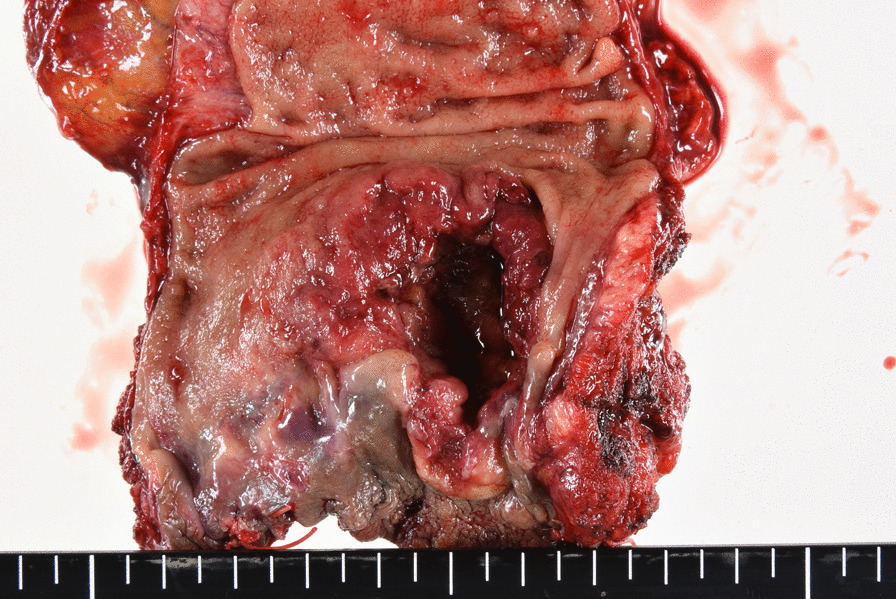
Gross findings. Ulcerative and infiltrative (type 3) tumor just above the dentate line (approximately 45 × 45 mm)

The patient was diagnosed with rectal cancer with a horseshoe kidney (T2N0M0, Stage I) and underwent surgery without neoadjuvant chemoradiotherapy. Laparoscopic abdominal perineal rectal resection was performed with a five-port conventional technique in which the sigmoid colon and the rectum were mobilized via a medial approach. The retroperitoneum was raised abdominally by the horseshoe kidney (Fig. 3a). Dissection between the sigmoid colon mesentery and the isthmus of the horseshoe kidney showed a clear release layer. During the operation, the left ureter, the left gonadal vessels, and the hypogastric nerve plexus were recognized on the surface of the isthmus and were preserved (Fig. 3b, c). The root of the aberrant renal artery was not visualized, whereas that of the IMA was identified on the temporal side of the renal isthmus, dividing immediately above the hypogastric nerve. Aided by 3D-CT angiography, D3 lymph node dissection was safely completed around the root of the IMA, avoiding intraoperative injury. The sigmoid colon and the rectum were dissected from the abdominal cavity to above the levator ani, and the tumor was excised from the incision wound around the perineum. The isthmus of the horseshoe kidney was bulging on the ventral side but was unharmed by the forceps during the operation. A sigmoid colostomy was constructed by the retroperitoneal route, and the perineal defect underwent primary closure. The operation time was 338 min, and the estimated blood loss was 200 mL. No immediate or delayed complications were observed. Gross findings of the resected specimen included an ulcerative and infiltrative (type 3) tumor (approximately 45 × 45 mm) (Fig. 4), and the pathological examination revealed that the tumor invaded beyond the muscle layer (T3) and identified 12 lymph nodes harvested from the specimen; however, it did not reveal any metastasis. Subsequently, the patient was classified as having Stage IIB (T3N0M0) rectal cancer.

The patient did not receive adjuvant chemotherapy. The patient has been stable and healthy for 28 months since she underwent the operation, without any evidence of rectal cancer recurrence.

## Discussion and conclusions

The horseshoe kidney malformation is caused by the fusion of the bilateral metanephric primordia during fetal life. Most horseshoe kidneys (about 95%) are fused at the lower pole, while the remaining 5% are fused at the upper pole. Their frequency of occurrence is approximately 1 in 400 and they are reportedly twice as common in men than in women. The horseshoe kidney exists lower than the normal kidney in the retroperitoneum because the constriction prevents the elevation of the kidney at the origin of the IMA. Although most horseshoe kidneys are asymptomatic, they may present with urinary tract infections, hydronephrosis, and calculus formation, and are often associated with the pelvic, ureteral, and vascular anomalies [1].

Twenty-nine cases of colorectal cancer with a horseshoe kidney were reported from 1983 to 2020 (15 men, 13 women), mostly in the Japanese literature, with only 2 cases in the English literature [2, 3]. The origin of the colorectal cancer was the sigmoid colon in 15 patients, the rectum in 10, the descending colon in 2, and the ascending colon in 1. Laparoscopic surgery was performed in 20 patients, while open laparotomy was conducted in the other 8 patients. To the best of our knowledge, this is the first study reporting the use of laparoscopic abdominal perineal rectal resection for lower rectal cancer.

In recent years, various studies reported laparoscopic colectomy to have superior perioperative results than open surgery [4–7]. In addition, given the standardization of surgical procedures and the emergence of useful devices, surgical applications have been expanding. In contrast, tactile sensation is reduced, visual field is restricted, and linear forceps are mainly used in laparoscopic surgery. Therefore, sufficient preoperative anatomical image evaluations and planning of a surgical strategy are essential for patients with anatomical variations to avoid intraoperative injury.

In recent years, remarkable advancements were observed in diagnostic imaging equipment, including the introduction of both multidetector-row CT (MDCT), which can capture thin slices and high-resolution images in a short time, and image construction software. These have made possible the development of effortless 3D-CT angiography with minimal invasiveness. When performing laparoscopic surgery for colorectal cancer, grasping and identifying the vascular branching anatomy as well as the anatomical vascular variations pre-operatively by 3D-CT angiography may be extremely useful. Indeed, it may greatly improve surgery quality by achieving optimal lymph node dissection, shortening surgery duration, and preventing complications through the identification of anatomical mutations and abnormal blood vessels [8–10]. 3D-CT angiography may also be useful for the imaging of left colorectal cancer with a horseshoe kidney, as previously performed in 10 of 28 cases. This allowed the assessment of the positional relationship with IMA, renal artery, and excess renal artery, which showed the latter to branch from the common iliac artery in 2 cases.

In surgeries for colorectal cancer coexisting with a horseshoe kidney, it is necessary to pay attention to the anatomy of the excess renal artery and vein, the ureter, the gonadal vessels, and the autonomic nerves.

The blood supply to a horseshoe kidney can be quite variable [1]. Arterial anomalies have been reported in more than 70% of patients with a horseshoe kidney. Of 21 cases described in detail in previous reports of colorectal cancer with a horseshoe kidney, 16 (including our patient) presented excess renal arteries. Kölln classified the common arterial blood supply to a horseshoe kidney into three types: (1) a single renal artery on each side originating from the aorta; (2) several renal arteries to each side and to the isthmus, all originating from the abdominal aorta; and (3) several renal arteries to each side and to the symphysis, some originating from the aorta and others originating from the common iliac, internal and external iliac, hypogastric or median sacral arteries [11]. The second type above described was observed in our patient. In addition, she also presented an excess renal arterial branch from the IMA [1], which could be injured during lymph node dissection around the IMA. Indeed, partial renal infarction may develop if this artery is damaged or an open conversion may be required due to bleeding during laparoscopic surgery. When ectopic blood vessels coming from the IMA were observed, we thought it necessary to preserve the aberrant excess renal artery from the IMA and dissect the superior rectal artery. The origin of the IMA prevents the elevation of the kidney. As the origin of the IMA may be covered by the renal isthmus, caution should be exercised when performing laparoscopic IMA root dissection. It is useful to perform 3D-CT angiography for identifying the route of the IMA and the excess renal artery and for understanding their relationship with the horseshoe kidney to prevent intraoperative injury.

Several cases of horseshoe kidney with anomalous inferior vena cava (IVC) have been described as pre-ischemic IVC or anatomical variations of the renal vein [12]. Therefore, during the operation for colorectal cancer with a horseshoe kidney, it is also necessary to verify the routes of the IVC and the renal vein. Generally, 3D-CT angiography conducted in patients with cancer with a horseshoe kidney mainly evaluates IMA, renal artery, and excess renal artery. However, we additionally confirmed the approximate relationship between the renal vein route and the horseshoe kidney using 3D-CT angiography to avoid intraoperative injury.

In patients with a horseshoe kidney, the ureter may originate high on the renal pelvis, lie laterally, and course downward, anterior to the isthmus [1]. All the reported cases showed the same route of the ureter on the ventral side of the horseshoe kidney. The ureter was preserved by following the usual procedure during the dissection of the mesentery of the colon.

In the reported cases, all the left gonadal vessels flowed into the left renal vein, ran in front of the horseshoe kidney like the ureter, and were preserved as in the ureter.

In most of the reported cases, as in our case, the bilateral lumbar splanchnic and superior hypogastric nerves run along the ventral side of the horseshoe kidney. However, there are also reports on autopsied cases where these nerves ran along the dorsal side of the kidney [3]. Therefore, reliably identifying and preserving these nerves during the operation is warranted.

Raising the horseshoe kidney on the ventral side reportedly results in the interruption of forceps manipulation during dissection around the IMA. Despite the limited space given the horseshoe kidney, we did not encounter such forceps limitations during peri-IMA dissection, sigmoid mesenteric recruitment, and pelvic surgery.

To the best of our knowledge, this is the third case report of laparoscopic left-sided colon cancer associated with a horseshoe kidney written in English. Similarly, this is the first report of an abdominal perineal rectal resection for lower rectal cancer in a patient with a horseshoe kidney. A horseshoe kidney is often associated with abnormalities in the blood vessels in the form of aberrant excess renal arteries, ureters, and/or nerves. It is possible to safely perform laparoscopic surgery for left-sided colon and rectal cancer by understanding the relationship between the vascular branching and the horseshoe kidney with preoperative 3D-CT angiography. It is important to maintain the same dissecting layer via a medial approach during the operation.

## Data Availability

The datasets supporting the conclusions of this article are included within the article.

## Abbreviations

3D: Three-dimensional
CT: Computed tomography
CTA: Computed tomography angiography
IMA: Inferior mesenteric artery
IVC: Inferior vena cava
